# The changing burden of gout in adults aged 70 and above based on the global burden of disease 2019

**DOI:** 10.3389/fpubh.2025.1455726

**Published:** 2025-02-06

**Authors:** Yang Yang, Zhong Liu

**Affiliations:** Department of Orthopedics, Xiangtan Central Hospital, Xiangtan, China

**Keywords:** global burden of disease, gout, older adults people, prevalence, public health

## Abstract

**Introduction:**

Gout is a significant global health issue, particularly among adults aged 70 and above. Understanding its epidemiological evolution and associated factors is crucial for guiding interventions and improving management.

**Methods:**

This study analyzed data from the Global Burden of Disease study to assess the prevalence and trends of gout among adults aged 70 and above from 1990 to 2019. We evaluated temporal trends and regional disparities by calculating the estimated annual percentage change (EAPCs).

**Results:**

Globally, the number of older adults individuals affected by gout increased from 5,316,210 cases in 1990 to 15,666,063 cases in 2019. It is noteworthy that the global burden of gout among the older adults continued to rise from 1990 to 2019, with an EAPC of 1.17 (95% CI: 1.02 to 1.32). In 2019, the prevalence rates among individuals aged 70–74, 75–79, 80–84, and 85 and above were 3,121.31 per 100,000, 3,437.17 per 100,000, 3,592.38 per 100,000, and 3,726.91 per 100,000, respectively. The regions with the highest prevalence rates of gout in 2019 were Australasia, High-income North America, and Southern Latin America, with rates of 8,500.73, 8,351.33, and 4,666.87 per 100,000, respectively. At the national level, some developed countries such as New Zealand, the United States of America, and Australia had the highest prevalence rates, reaching 8,893.74, 8,508.06, and 8,427.94 per 100,000, respectively. It is noteworthy that regions with higher levels of Socio-Demographic Index tended to have relatively higher burden of gout among the older adults, and the prevalence rates varied across different regions and age groups.

**Conclusion:**

The study underscores the persistent burden of gout among the older adults, emphasizing the need for targeted interventions to address this issue and improve public health outcomes in this demographic.

## Introduction

Gout, formerly labeled the “disease of kings,” has transitioned from a malaise tied to wealth to one impacting a wider demographic, particularly the older adults (1, 2). This longstanding condition, caused by the buildup of monosodium urate crystals in joints, has seen a global rise in prevalence, paralleling the increase in non-communicable diseases. In addition to its health impact, gout contributes to healthcare costs and is linked to renal and cardiovascular diseases. It also causes productivity losses due to frequent flare-ups (3). Age emerges as a pivotal factor in gout risk, with incidence rates escalating notably after 40 and peaking among those aged 70 and above (4). This demographic shift is propelled by a intricate interplay of age-related physiological changes, encompassing reduced renal uric acid excretion and alterations in purine metabolism, compounded by the cumulative burden of prevalent comorbidities in older adults, such as hypertension, diabetes, and cardiovascular disease (5). Age is an important factor, but other confounding variables like lifestyle, comorbidities (such as obesity or diet), could also significantly influence gout risk. These factors, however, were not the focus of this analysis and could be further explored in future studies.

Moreover, lifestyle elements like diet, alcohol intake, and sedentary habits, once synonymous with affluent lifestyles, have become widespread across diverse socio-economic strata, exacerbating the burden of gout among older populations worldwide (6). Despite its pervasive impact, gout in older adults remains often overlooked and undertreated, resulting in substantial morbidity, diminished quality of life, and heightened healthcare utilization (7). The dearth of comprehensive epidemiological data on gout burden, particularly among those aged 70 and above, impedes efforts to devise targeted interventions and allocate resources effectively to tackle this burgeoning public health challenge (7, 8).

Leveraging the comprehensive dataset of the Global Burden of Disease (GBD) project, renowned for reshaping our understanding of global health dynamics, our study aims to elucidate the trajectory of gout prevalence and its impact on this demographic (9, 10). By analyzing GBD data from 1990 to 2019, spanning a wide range of countries and regions, we seek to uncover trends in gout incidence among older adults. Through this analysis, we aim to provide valuable insights to guide evidence-based interventions aimed at alleviating the global burden of gout and improving health outcomes for individuals aged 70 and above.

## Methods

### Data sources

The utilization of the Global Health Data Exchange Query Tool facilitated access to the GBD database (https://ghdx.healthdata.org/gbd-2019) (10). In this investigation, a meticulous evaluation was conducted on 369 diseases and injuries, alongside an analysis of 87 risk factors (10, 11). Subsequent to this initial phase, the tool was then employed to retrieve data pertaining to gout incidence rates from a span of 204 countries and 21 regions, covering the period from 1990 to 2019 (12, 13). The categorization of these 21 regional groupings of countries was based on considerations of geographic proximity and shared epidemiological characteristics (14, 15).

Through the utilization of Socio-demographic Index (SDI) data, a segmentation of various regions and countries was performed, delineating them into categories of low, low-middle, middle, middle-high, and high-SDI (16). The primary aim was to explore the potential correlation between SDIs and the prevalence of gout among individuals aged 70 years and older (17). Our comprehensive data collection efforts were inclusive of individuals from both genders and spanned across four distinct age groups: 70–74 years, 75–79 years, 80–84 years, and 85 years and older (18). Notably, this research is grounded upon publicly available databases and does not necessitate ethical approval.

### Statistical analysis

This study was conducted to ascertain the burden of gout among individuals aged 70 years and older across diverse regions and countries within the GBD 2019, meticulously determining a 95% uncertainty interval (UI) for each parameter (19). Utilizing the incidence rate per 100,000 population as a foundation for population-size-based rates, the investigation delved into the trends in incidence rates over a specified period, employing estimated annual percentage changes (EAPCs) for characterization (20). We selected the EAPC model for its simplicity and suitability in identifying broad, long-term trends in gout incidence, aligning with the study’s aim to provide a population-level overview. Given the chronic nature of gout, our analysis excludes non-linear trends and seasonality, focusing on overall trends across regions and time. However, alternative methods, such as multivariate regression models or subgroup analysis, may offer deeper insights into the impact of confounding variables, which could be considered in future research. Employing a joint regression model, regression analysis was undertaken to dissect the disease burden. To calculate the EAPC, a logarithmic linear regression model was adopted, with the natural logarithm of the incidence rate (y) as the dependent variable and the calendar year (x) as the independent variable, following the formula:


y=α+βx+ε

$$\text{EAPC}\;=\;100\%\times \left(e^{\beta }–1\right)$$

Notably, trends indicating an increase in burden were depicted by lower limits of the EAPC and its 95% CI exceeding 0, while trends indicating a decrease were illustrated by upper limits of the EAPC and its 95% CI dipping below 0. Furthermore, Pearson correlation analysis was conducted to explore the association between the EAPC in 1990 and the incidence rate of gout among individuals aged 70 years and older (21). Similarly, correlations between the EAPC in 2019 and the SDI were investigated. Additionally, the correlation between gout incidence rates and SDI for all countries was examined. The obtained correlation coefficient (*ρ*) and corresponding *p*-values were used to evaluate the strength and significance of these associations.

## Results

### Global estimates of gout prevalence among older adults aged 70 and above

**Figure 1:**
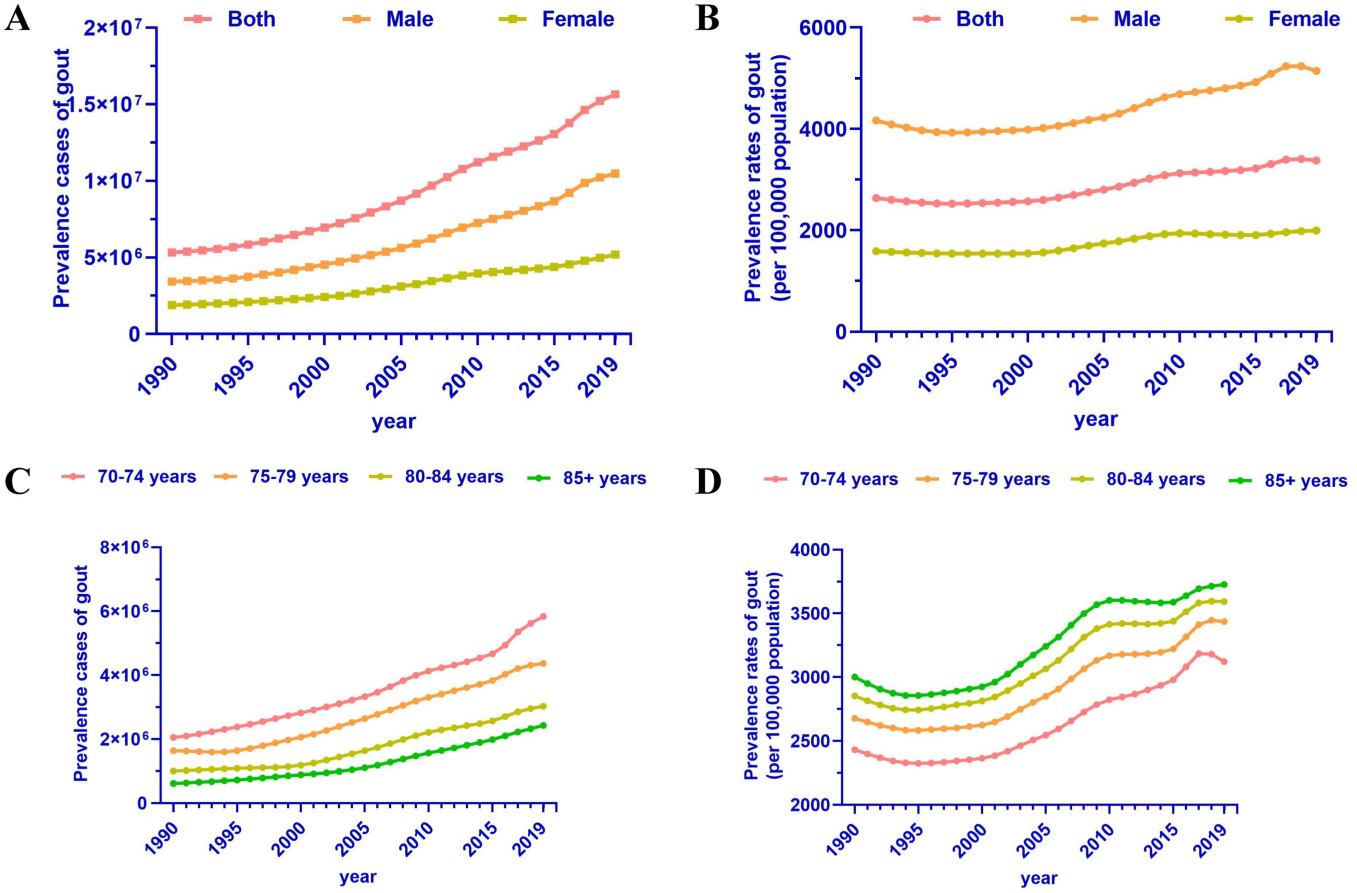
Trends in gout prevalence in adults aged 70 and above, 1990 to 2019. **(A)** Prevalence cases of gout by sex. **(B)** Prevalence rates of gout by sex. **(C)** Prevalence cases of gout by age group. **(D)** Prevalence rates of gout by age group.

Globally, the prevalence and trends of gout among individuals aged 70 and above from 1990 to 2019 are illustrated in Figure 1. As depicted in Figure 1A, the number of cases increased from 5,316,210 in 1990 to 15,666,063 in 2019, approximately tripling over the period. It is noteworthy that the number of cases in males was twice that of females, with 10,469,254 cases in males and 5,196,809 cases in females in 2019 (Table 1). Considering the changing global demographic structure, the prevalence of gout among individuals aged 70 and above (including males and females) has continuously increased by 1.17 (95% CI: 1.02 to 1.32) over the 30-year period (Figure 1B and Table 1). This underscores the growing burden of gout among the older adults population globally, indicative of improvements in living standards. Additionally, hyperuricemia, a key factor in gout development, has significant implications for health, contributing to an increased risk of renal and cardiovascular diseases, as well as other metabolic disorders (22).

**Table 1:** The global prevalence rates and their change trends of gout aged 70+ from 1990 to 2019.

Characteristic	Cases_1990	Cases_2019	Cases_change	Rates_1990	Rates_2019	EAPC_CI
Global	5,316,210 (3,777,893 to 7,119,246)	15,666,063 (11,472,118 to 20,377,053)	1.95 (1.86 to 2.07)	2637.70 (1874.45 to 3532.30)	3378.67 (2474.17 to 4394.68)	1.17 (1.02 to 1.32)
Sex						
Female	1,892,599 (1,326,232 to 2,522,938)	5,196,809 (3,800,303 to 6,770,118)	1.75 (1.65 to 1.89)	1586.22 (1111.54 to 2114.52)	1996.76 (1460.18 to 2601.28)	1.09 (0.93 to 1.26)
Male	3,423,611 (2,438,770 to 4,579,369)	10,469,254 (7,679,209 to 13,669,666)	2.06 (1.96 to 2.18)	4163.35 (2965.71 to 5568.84)	514,677 (3775.16 to 6720.12)	1.05 (0.89 to 1.21)
Age						
70–74 aged	2,054,548 (1,357,009 to 2,910,296)	5,839,571 (3,948,444 to 8,154,114)	1.84 (1.76 to 1.94)	2431.03 (1605.67 to 3443.58)	3121.31 (2110.48 to 4358.45)	1.18 (1.02 to 1.33)
75 79 aged	1,641,447 (1,125,971 to 2,262,237)	4,367,056 (3,076,023 to 5,862,878)	1.66 (1.59 to 1.76)	2677.32 (1836.54 to 3689.88)	3437.17 (2421.04 to 4614.48)	1.11 (0.98 to 1.25)
80–84 aged	1,004,867 (646,362 to 1,492,787)	3,032,776 (2,008,923 to 4,427,786)	2.02 (1.93 to 2.14)	2853.14 (1835.23 to 4238.50)	3592.38 (2379.61 to 5244.80)	1.1 (0.97 to 1.24)
85+ aged	615,348 (433,721 to 850,091)	2,426,660 (1,759,024 to 3,272,478)	2.94 (2.8 to 3.11)	3001.08 (2115.28 to 4145.94)	3726.91 (2701.54 to 5025.94)	1.1 (0.95 to 1.25)
SDI regions						
High SDI	2,315,548 (1,662,110 to 3,092,105)	6,533,153 (5,011,397 to 8,220,821)	1.82 (1.64 to 2.06)	3516.10 (2523.87 to 4695.28)	5090.64 (3904.88 to 6405.67)	1.83 (1.61 to 2.05)
High-middle SDI	1,272,263 (895,506 to 1,700,001)	3,538,606 (2,525,011 to 4,745,667)	1.78 (1.71 to 1.86)	2293.45 (1614.29 to 3064.52)	2974.34 (2122.37 to 3988.92)	1.1 (0.97 to 1.24)
Middle SDI	1,022,560 (727,511 to 1,375,121)	3,593,678 (2,530,865 to 4,836,968)	2.51 (2.41 to 2.62)	2260.40 (1608.18 to 3039.75)	2867.30 (2019.31 to 3859.29)	1.06 (0.92 to 1.19)
Low-middle SDI	496,819 (350,967 to 672,969)	1,493,929 (1,057,420 to 2,007,544)	2.01 (1.93 to 2.09)	1970.01 (1391.67 to 2668.49)	2188.50 (1549.05 to 2940.92)	0.4 (0.36 to 0.44)
Low SDI	206,925 (146,631 to 278,525)	501,469 (355,282 to 671,464)	1.42 (1.35 to 1.5)	2144.05 (1519.32 to 2885.94)	2225.08 (1576.43 to 2979.37)	0.09 (0.07 to 0.12)
Location						
Andean Latin America	10,782 (7,631 to 14,429)	41,579 (29,893 to 55,265)	2.86 (2.68 to 3.08)	1056.18 (747.53 to 1413.46)	1323.19 (951.30 to 1758.73)	0.84 (0.81 to 0.87)
Australasia	85,059 (62,216 to 114,513)	281,986 (210,200 to 370,429)	2.32 (2.03 to 2.64)	5836.54 (4269.14 to 7857.64)	8500.73 (6336.67 to 11166.94)	1.46 (1.34 to 1.58)
Caribbean	14,153 (9,984 to 19,180)	34,811 (24,958 to 46,871)	1.46 (1.35 to 1.58)	949.73 (669.97 to 1287.06)	1129.44 (809.76 to 1520.74)	0.64 (0.62 to 0.67)
Central Asia	39,190 (27,595 to 52,749)	55,731 (39,274 to 74,742)	0.42 (0.36 to 0.48)	1761.36 (1240.26 to 2370.78)	1989.65 (1402.11 to 2668.36)	0.51 (0.48 to 0.54)
Central Europe	112,698 (78,806 to 154,300)	221,356 (157,670 to 298,490)	0.96 (0.91 to 1.03)	1436.20 (1004.29 to 1966.36)	1583.46 (1127.89 to 2135.23)	0.44 (0.38 to 0.5)
Central Latin America	25,494 (17,850 to 34,864)	93,156 (66,360 to 125,447)	2.65 (2.55 to 2.76)	627.15 (439.10 to 857.65)	715.15 (509.44 to 963.05)	0.7 (0.55 to 0.84)
Central Sub-Saharan Africa	20,907 (14,638 to 28,496)	47,033 (32,747 to 63,614)	1.25 (1.09 to 1.43)	2498.48 (1749.35 to 3405.48)	2410.26 (1678.18 to 3260.00)	−0.16 (−0.23 to −0.08)
East Asia	1,061,024 (751,282 to 1,441,036)	4,205,637 (2,952,393 to 5,679,490)	2.96 (2.8 to 3.13)	2677.71 (1896.01 to 3636.75)	3754.38 (2635.61 to 5070.10)	1.6 (1.4 to 1.81)
Eastern Europe	268,899 (188,474 to 365,174)	427,184 (302,310 to 573,129)	0.59 (0.54 to 0.65)	1775.42 (1244.41 to 2411.09)	2087.77 (1477.47 to 2801.04)	0.68 (0.63 to 0.72)
Eastern Sub-Saharan Africa	79,423 (56,371 to 106,984)	179,418 (126,923 to 239,819)	1.26 (1.19 to 1.33)	2521.75 (1789.83 to 3396.83)	2638.86 (1866.78 to 3527.24)	0.14 (0.12 to 0.17)
High-income Asia Pacific	358,118 (249,805 to 479,677)	1,198,965 (863,685 to 1,588,946)	2.35 (2.21 to 2.52)	3179.12 (2217.59 to 4258.25)	3606.10 (2597.68 to 4779.03)	0.5 (0.48 to 0.53)
High-income North America	1,072,781 (777,464 to 1,429,993)	3,378,574 (2,716,986 to 4,116,484)	2.15 (1.8 to 2.63)	4593.51 (3329.00 to 6123.05)	8351.33 (6715.98 to 10175.34)	3.29 (2.75 to 3.84)
North Africa and Middle East	170,757 (120,603 to 229,661)	507,252 (357,391 to 679,606)	1.97 (1.87 to 2.07)	2282.28 (1611.94 to 3069.58)	2598.91 (1831.09 to 3481.96)	0.39 (0.33 to 0.44)
Oceania	3,526 (2,466 to 4,801)	9,174 (6,454 to 12,513)	1.6 (1.46 to 1.79)	3243.18 (2268.12 to 4415.93)	3541.46 (2491.49 to 4830.22)	0.31 (0.28 to 0.34)
South Asia	413,727 (291,332 to 559,608)	1,414,244 (1,003,246 to 1,904,109)	2.42 (2.32 to 2.53)	1889.29 (1330.37 to 2555.45)	2045.94 (1451.36 to 2754.62)	0.22 (0.18 to 0.25)
Southeast Asia	272,663 (194,066 to 366,451)	814,479 (574,546 to 1,095,299)	1.99 (1.9 to 2.08)	2495.23 (1775.96 to 3353.51)	2932.30 (2068.49 to 3943.32)	0.64 (0.61 to 0.66)
Southern Latin America	99,524 (70,464 to 134,368)	244,138 (175,368 to 328,659)	1.45 (1.32 to 1.6)	3783.25 (2678.58 to 5107.78)	4666.87 (3352.28 to 6282.54)	0.74 (0.68 to 0.8)
Southern Sub-Saharan Africa	38,159 (26,986 to 50,957)	79,753 (56,211 to 107,130)	1.09 (1.03 to 1.15)	2867.78 (2028.08 to 3829.58)	3008.05 (2120.13 to 4040.62)	0.15 (0.09 to 0.21)
Tropical Latin America	41,150 (28,757 to 55,726)	157,002 (112,392 to 210,523)	2.82 (2.67 to 2.98)	946.78 (661.64 to 1282.13)	1173.53 (840.08 to 1573.58)	0.8 (0.76 to 0.84)
Western Europe	1,036,249 (742,703 to 1,393,819)	2,088,578 (1,527,132 to 2,773,769)	1.02 (0.94 to 1.1)	2773.70 (1987.97 to 3730.80)	3282.87 (2400.38 to 4359.87)	0.81 (0.72 to 0.89)
Western Sub-Saharan Africa	91,928 (64,870 to 123,285)	186,013 (131,443 to 250,463)	1.02 (0.98 to 1.07)	2308.31 (1628.87 to 3095.67)	2374.50 (1677.90 to 3197.21)	0.01 (−0.13 to 0.14)

Data in parentheses are 95% uncertainty intervals for cases and rates, and 95% confidence intervals for EAPC.

We conducted a detailed analysis among older adults individuals in different age groups. From 1990 to 2019, the number of gout patients in the 70–74 age group was significantly higher than in other age groups, followed by the 75–79 age group, 80–84 age group, and 85+ age group (Figure 1C). However, although the number of patients aged 70–74 was the highest, the prevalence rate was the opposite, possibly related to the population base of the corresponding age group. Specifically, the prevalence rates of gout among older adults individuals aged 70–74, 75–79, 80–84, and 85+ were 3121.31 per 100,000, 3437.17 per 100,000, 3592.38 per 100,000, and 3726.91 per 100,000, respectively (Figure 1D and Table 1). Regardless of the age group, the trend of gout among the older adults has been on the rise (EAPC between 1 and 1.18), indicating an increasing burden of gout among the older adults population (23). This highlights the need for attention to evidence-based interventions.

### Regional differences of gout prevalence among older adults aged 70 and above

**Figure 2:**
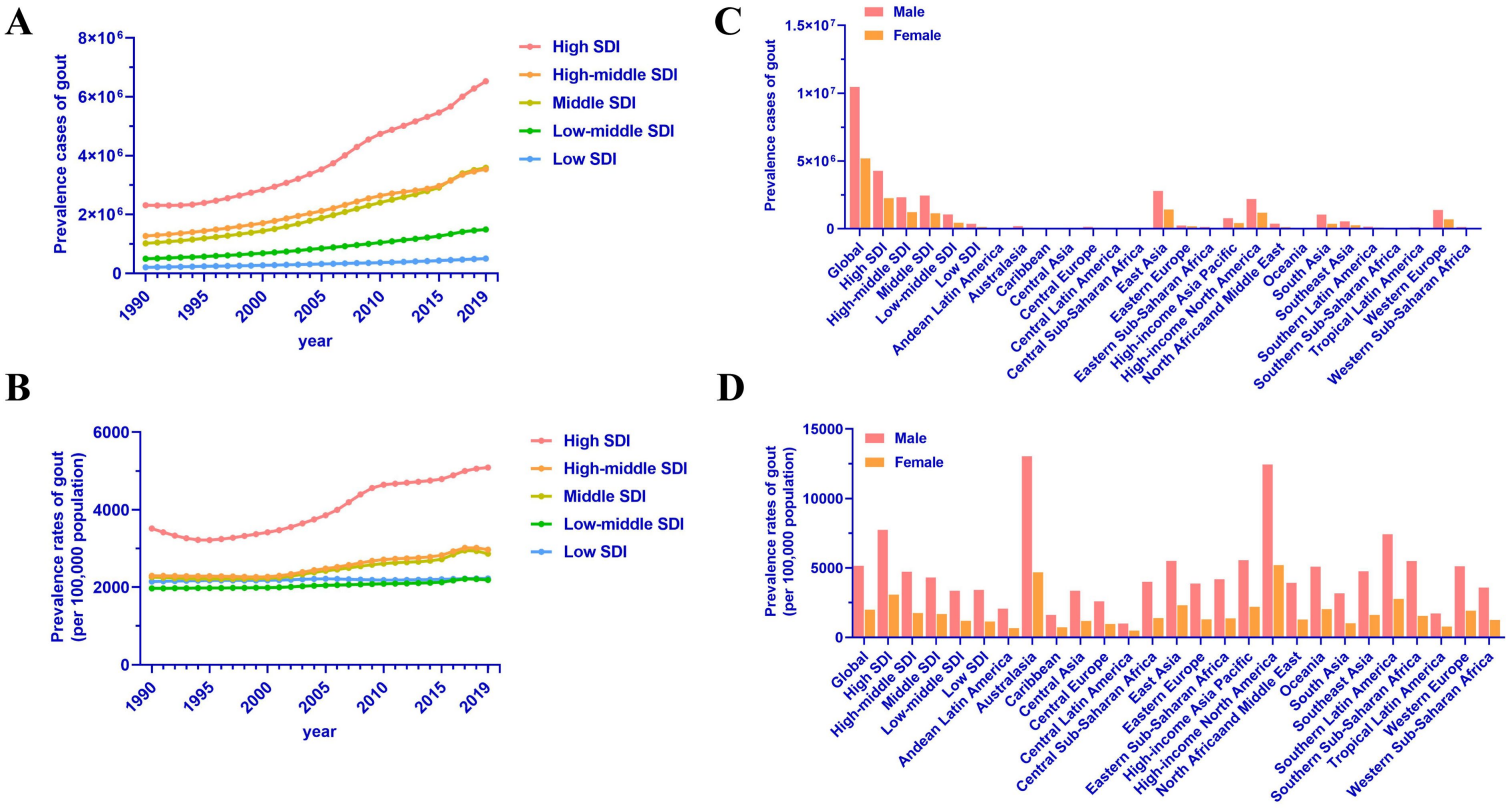
Regional differences in gout prevalence in adults aged 70 and above. **(A)** Prevalence cases of gout by SDI regions. **(B)** Prevalence rates of gout by SDI regions. **(C)** Prevalence cases of gout in 2019 in low to high SDI and 21 GBD regions. **(D)** Prevalence rates of gout in 2019 in low to high SDI and 21 GBD regions.

Within the global landscape, significant disparities exist in both the absolute number and prevalence of gout cases among individuals aged 70 and above, influenced by varying levels of socioeconomic development and geographical locations. Generally, in regions with high SDI, the burden of gout has markedly increased, with the number of cases rising from 2,315,548 in 1990 to 6,533,153 in 2019, representing a growth rate of 1.82 (Figure 2A and Table 1). However, it’s notable that in regions with higher SDI, the prevalence of gout among individuals aged 70 and above is also higher, while areas with lower SDI exhibit lower prevalence rates (Figure 2B). For instance, in High SDI regions, the prevalence reached 5,090.64 per 100,000 people in 2019, whereas in Low SDI regions, it was 2,225.08 per 100,000 people (Figure 2B and Table 1). Across High SDI, High-middle SDI, Middle SDI, Low-middle SDI, and Low SDI regions, the trends of gout burden, as indicated by EAPCs, were 1.83 (95% CI: 1.61 to 2.05), 1.1 (95% CI: 0.97 to 1.24), 1.06 (95% CI: 0.92 to 1.19), 0.4 (95% CI: 0.36 to 0.44), and 0.09 (95% CI: 0.07 to 0.12), respectively (Table 1). This illustrates that the higher quality of life in High SDI regions has contributed to the increased burden of gout.

There are significant disparities in the prevalence of gout among individuals aged 70 and above across different regions. In 2019, the number of cases in 21 GBD regions is illustrated in Figure 2C. The regions with the highest prevalence rates were Australasia (8,500.73 per 100,000), High-income North America (8,351.33 per 100,000), and Southern Latin America (4,666.87 per 100,000). Specifically, the prevalence rates for males were 13,037.29 per 100,000, 12,456.31 per 100,000, and 7,427.33 per 100,000, respectively, while for females, the rates were 5,195.93 per 100,000, 4,684.13 per 100,000, and 2,767.55 per 100,000 (Figure 2D). It is noteworthy that from 1990 to 2019, the regions with the most rapid growth in gout burden among the older adults were High-income North America, East Asia, and Australasia, with EAPCs of 3.29 (95% CI: 2.75 to 3.84), 1.6 (95% CI: 1.4 to 1.81), and 1.46 (95% CI: 1.34 to 1.58), respectively (Table 1).

According to our analysis, we found that the prevalence of gout among the older adults is relatively low in Tropical Latin America, the Caribbean, and Central Latin America, with incidence rates of 1,173.53 per 100,000, 1,129.44 per 100,000, and 715.15 per 100,000, respectively (Table 1). Specifically, the prevalence rates for males were 1,712.33 per 100,000, 1,618.82 per 100,000, and 996.89 per 100,000, while for females, the rates were 777.53 per 100,000, 726.98 per 100,000, and 486.03 per 100,000 (Table 1). Interestingly, among these 21 geographical regions, only Central Sub-Saharan Africa exhibited a decreasing burden of gout, with an EAPC of −0.16 (95% CI: −0.23 to −0.08).

### National differences of gout prevalence among older adults aged 70 and above

**Figure 3:**
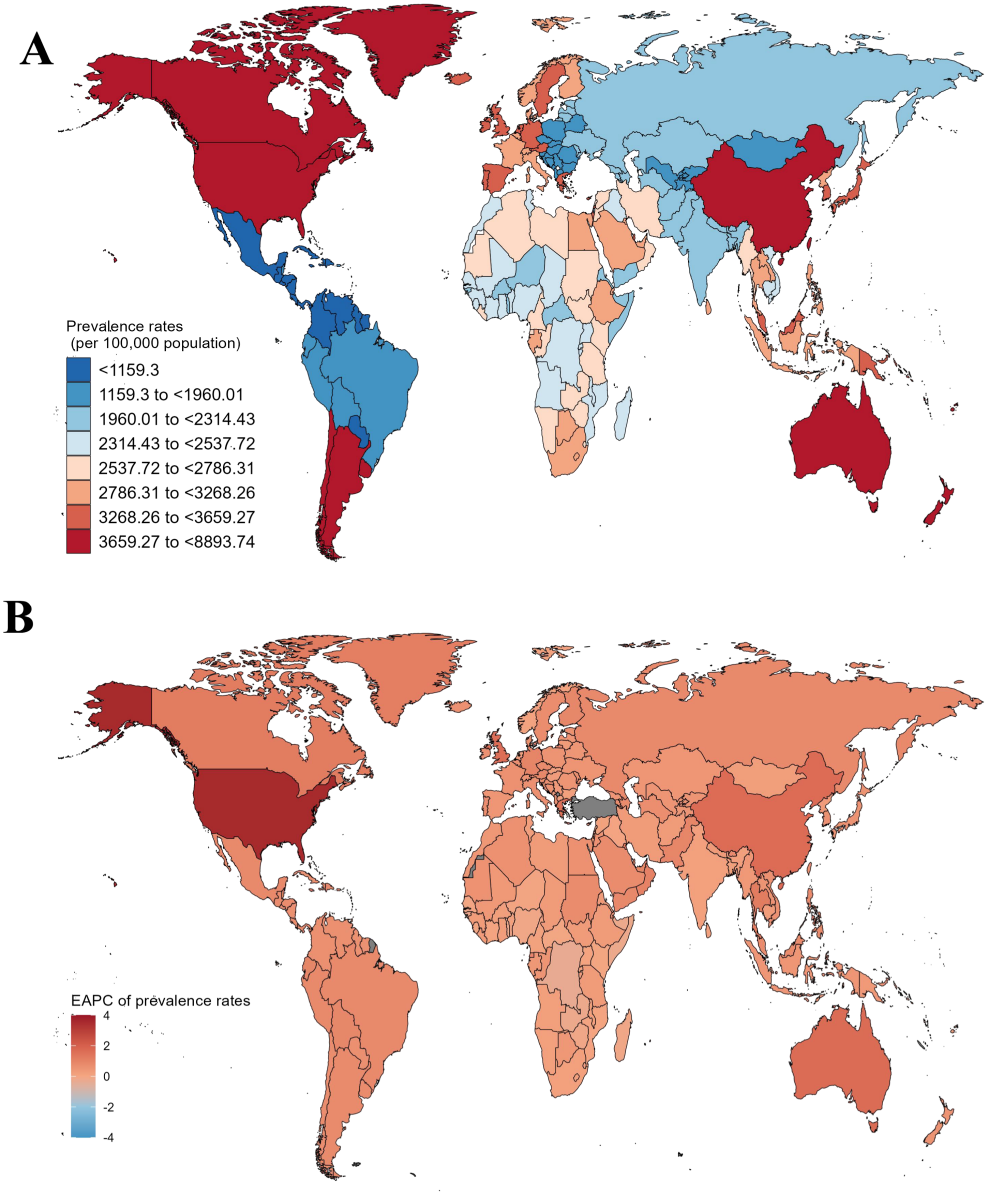
The global prevalence rates of gout among individuals aged 70 and above, as well as their changing trends from 1990 to 2019, vary across different countries and regions. **(A)** Prevalence rates of gout in 204 countries in 2019. **(B)** The EAPC of gout prevalence rates in 204 countries from 1990 to 2019.

Due to regional disparities, there are significant variations in the prevalence of gout among individuals aged 70 and above across different countries, reflecting the influence of various factors. At the national level, among the 204 countries analyzed, China, the United States of America, and India had the highest absolute numbers of cases in 2019, with 4,041,719, 3,059,118, and 1,156,386 cases, respectively (Supplementary Table S1). This could be attributed to the large population sizes of these countries. When considering the prevalence per 100,000 individuals, we found that some developed countries such as New Zealand, the United States of America, and Australia had the highest prevalence rates, reaching 8,893.74 per 100,000, 8,508.06 per 100,000, and 8,427.94 per 100,000, respectively (Figure 3A and Supplementary Table S1). In contrast, countries with lower levels of economic development, such as Guatemala, Honduras, and Nicaragua, had very low rates of gout prevalence, with only 604.19 per 100,000, 618.75 per 100,000, and 618.93 per 100,000 individuals, respectively (Figure 3A and Supplementary Table S1).

It is noteworthy that over the past 30 years, Togo, the Democratic Republic of the Congo, and Zambia have effectively managed the burden of gout among the older adults, showing the most significant declines. Their EAPCs were − 0.34 (95% CI: −0.38 to −0.3), −0.34 (95% CI: −0.43 to −0.24), and −0.23 (95% CI: −0.27 to −0.19), respectively (Figure 3B and Supplementary Table S1). In contrast, China, Australia, and the United States of America have experienced a continuous increase in the burden of gout, with EAPCs of 1.66 (95% CI: 1.42 to 1.91), 1.68 (95% CI: 1.51 to 1.85), and 3.55 (95% CI: 2.93 to 4.18), respectively (Figure 3B and Supplementary Table S1). In fact, these countries have also undergone rapid economic development in recent decades, suggesting that multi-country cooperation in healthcare strategies and public health initiatives could help mitigate the growing burden of gout (24).

### Correlations between EAPC, prevalence rates, and SDI in gout prevalence among older adults aged 70 and above

**Figure 4:**
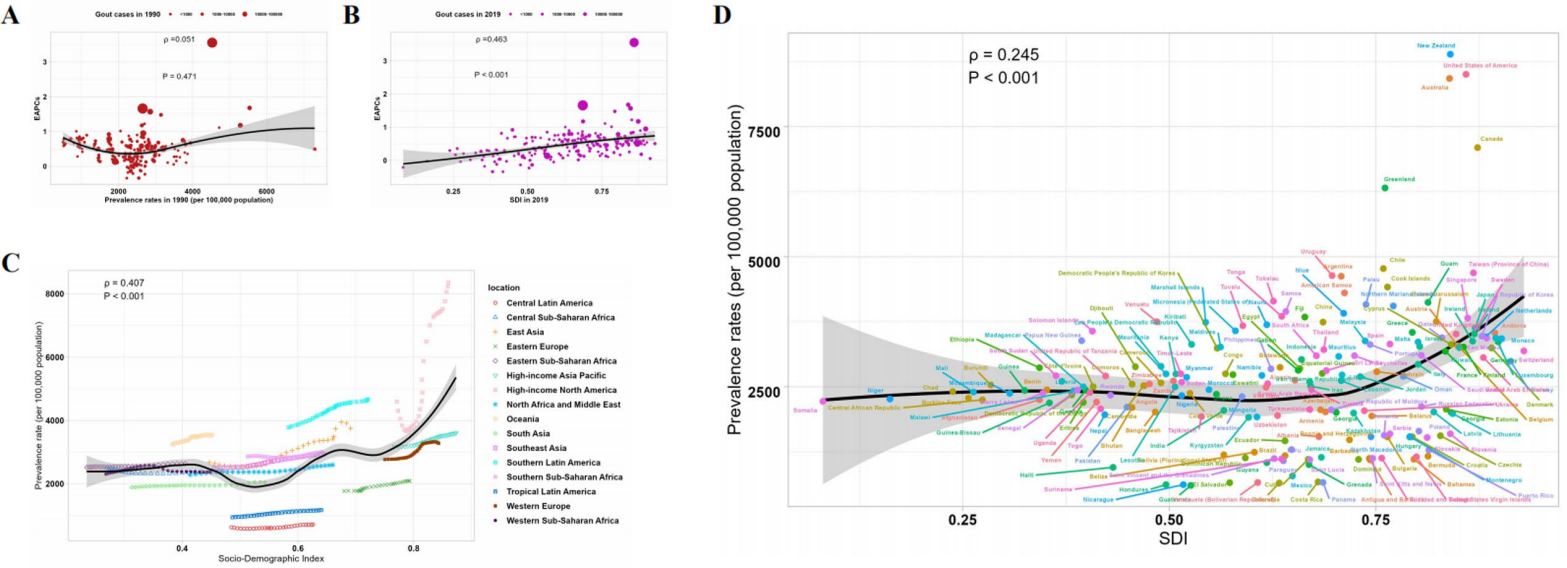
Correlation of EAPC, prevalence, and SDI of gout prevalence in adults aged 70 and above. **(A)** The relationship between EAPC and prevalence rates in 1990. **(B)** The association between EAPC and SDI in 2019. **(C)** The correlation between prevalence and SDI at the regional level. **(D)** The relationship between prevalence and SDI at the national level.

Expanding upon our initial epidemiological inquiry, we uncovered a noteworthy correlation between gout prevalence among the older adults and the SDI across diverse regions. This emphasizes the intricate relationship between socioeconomic factors and the incidence of this disease, shedding light on their complex interplay. Subsequent correlation analysis focused on the trends of gout prevalence among the older adults. Figure 4A illustrates that while there was no direct correlation between the trends represented by EAPC values and the prevalence in 1990 (*ρ* = 0.051, *p* = 0.471), a robust positive correlation emerged with higher SDI levels (*ρ* = 0.463, *p* < 0.001) (Figure 4B). Further examination of SDI levels across different regions revealed a compelling trend: regions with higher SDI levels tended to exhibit relatively higher prevalence rates (*ρ* = 0.407, *p* < 0.001) (Figure 4C). Similarly, at the national level, this correlation appeared evident (*ρ* = 0.245, *p* < 0.001) (Figure 4D), potentially attributed to the diverse socioeconomic conditions within each country. These findings underscore the presence of complex interactions among various factors influencing the prevalence of gout among the older adults, emphasizing the need for nuanced, context-specific approaches to address this health challenge.

## Discussion

The pathogenesis of gout is a complex process involving disturbances in uric acid metabolism, genetic factors, lifestyle, and the interactions of various chronic diseases (25, 26). Recent research indicates that in addition to elevated uric acid levels, multiple factors such as inflammation, aberrant immune responses, and lifestyle-related conditions (e.g., obesity, dietary habits) play significant roles in the onset and progression of gout. However, these factors were not comprehensively included in the current analysis, and future studies should explore their impact in more detail (25). For example, emerging studies suggest that inflammatory mediators play crucial roles during gout flares and may serve as new therapeutic targets (2, 27, 28). Additionally, genetic factors are considered important contributors to gout onset, particularly among the older adults population, where genetic susceptibility may exacerbate the development and worsening of gout (29). However, with socioeconomic development and lifestyle changes, gout has transitioned from being a disease primarily associated with affluent individuals to one affecting a broader population, especially the older adults (29, 30). This transition reflects global trends of rising non-communicable disease prevalence and the impact of global population aging (31). Despite the increasing prominence of gout among the older adults population, clinical practice often overlooks and inadequately treats gout in this demographic, leading to serious health issues and wastage of medical resources.

In this study, we utilized the comprehensive dataset from the GBD project to analyze the trends in gout prevalence among individuals aged 70 and above worldwide. Our analysis showed a consistent increase in gout prevalence among the older adults globally, particularly in developed countries and regions. This trend may be influenced by unaccounted confounding factors such as lifestyle changes, rising obesity rates, and associated comorbidities, which were not explicitly controlled for in this study. This increase is likely associated with improvements in living standards, changes in dietary patterns, rising obesity rates, and increasing prevalence of chronic diseases. However, the current analysis did not employ advanced statistical techniques, such as multivariate regression or stratified analysis, which could have better controlled for these confounding factors and provided more nuanced insights. Specifically, in some developed countries, there is a noticeable upward trend in gout prevalence, possibly due to factors such as high-sodium, high-fat diets, alcohol consumption, and lack of physical activity. It is noteworthy that in some low and middle-income countries and regions, the prevalence of gout is relatively low, which may be attributed to traditional dietary patterns, rural lifestyles, and insufficient healthcare resources (32, 33). Additionally, underexcretion of uric acid is a key factor in gout and contributes to its increasing prevalence. URAT-1 inhibitors offer promising treatments by improving uric acid excretion and reducing flare-ups. These advancements could help reduce the growing burden of gout, particularly in countries with rising prevalence (34).

In recent years, significant progress has been made in gout research, deepening our understanding of its pathogenesis and providing new directions and strategies for its prevention and treatment (25). Recent studies have shown that gout onset is not only associated with abnormalities in uric acid metabolism but also involves the activation of inflammatory mediators, genetic susceptibility, and lifestyle factors (32, 33). Among these, emerging research has identified inflammatory mediators as key players during gout flares, serving as novel therapeutic targets (35). By investigating the changes in inflammatory mediators such as IL-1β and IL-6 during gout flares, scientists have found a significant association between their increase and joint pain and inflammatory responses in gout patients (36). Therefore, interventions targeting inflammatory mediators, such as the use of IL-1β inhibitors, have become one of the new strategies for gout treatment.

Furthermore, recent genetic studies have highlighted the significant role of genetic factors in the onset of gout (37). Scientists have found that mutations in specific genes are closely associated with the risk of developing gout, particularly among the older adults population (38). The discovery of these genetic susceptibility genes provides new insights into personalized prevention and treatment strategies for gout, aiding in better identification and intervention for high-risk individuals (38). The proportion of the older adults population in each country influences the burden of gout. Countries with a higher percentage of older adults individuals tend to have a higher prevalence of gout due to the increased risk associated with aging. This demographic shift highlights the need for targeted healthcare strategies for aging populations. Additionally, recent epidemiological studies have revealed the importance of lifestyle factors in the onset of gout. Factors such as obesity, high-sodium diets, alcohol consumption, and lack of exercise are closely related to the risk of developing gout (39). Therefore, lifestyle interventions such as weight control, improving dietary patterns, limiting alcohol intake, and increasing physical activity are considered essential measures for preventing and managing gout, helping to reduce the incidence of gout and alleviate symptoms.

### Strengths and limitations

Although our study offers important insights into global gout trends among the older adults population aged 70 and above, it has several notable limitations. First, the use of a relatively simple statistical model, such as the EAPC, did not allow for the adjustment of key confounding factors, including comorbid conditions (e.g., diabetes, cardiovascular diseases) and lifestyle factors (e.g., diet, alcohol consumption, physical activity), which are known to influence gout prevalence. Second, data accuracy may be affected by variations in reporting practices and potential underreporting, particularly in regions with limited healthcare infrastructure. These factors could result in an incomplete understanding of the underlying drivers of the observed trends. Additionally, the use of a simple EAPC model without adjusting for potential confounding variables may have led to an incomplete understanding of the factors contributing to the increase in gout prevalence (15, 23). Nonetheless, our study serves as a foundation for future research and intervention efforts, emphasizing the need for improved data quality and the consideration of broader age ranges and geographical factors to enhance the effectiveness of interventions.

## Conclusion

In summary, gout is on the rise worldwide, especially among the older adults, notably males, with prevalence peaking in those aged 70–74. Regional and national disparities are evident, with higher rates in more developed areas. While this study highlights important trends, future research should consider more complex models to account for confounding factors like comorbidities and lifestyle. These findings emphasize gout as a global health concern, guiding more targeted prevention and intervention strategies.

## Data Availability

The original contributions presented in the study are included in the article/Supplementary material, further inquiries can be directed to the corresponding author/s.
