# Supplementary material for: The changing burden of gout in adults aged 70 and above based on the global burden of disease 2019
Source: Front Public Health. 2025 Feb 6;13:1455726. doi: 10.3389/fpubh.2025.1455726 (PMC11840442; doi:10.3389/fpubh.2025.1455726)
Supplement: Supplementary file 1 [file Data_Sheet_1.pdf]

**Supplementary Table S1.** Prevalence of gout aged 70+ in 1990 and 2019 for both sexes and all locations, with EAPC from 1990 and 2019.

| location | Cases_1990 | Cases_2019 | Rates_1990 | Rates_2019 | EAPC_CI |
|---|---|---|---|---|---|
| Afghanistan | 6975(4911 to 9630) | 11205(7829 to 15558) | 2129.22(1499.06 to 2939.79) | 2252.55(1573.91 to 3127.67) | 0.27(0.21 to 0.33) |
| Albania | 1361(968 to 1850) | 4091(2883 to 5555) | 1348.93(959 to 1834.14) | 1541.91(1086.55 to 2093.5) | 0.6(0.56 to 0.64) |
| Algeria | 13403(9244 to 18280) | 41940(29009 to 57423) | 2206.07(1521.61 to 3008.79) | 2627.45(1817.36 to 3597.38) | 0.59(0.56 to 0.62) |
| American Samoa | 35(24 to 47) | 97(68 to 132) | 3862.23(2691.18 to 5227.57) | 4305.36(3020.81 to 5863.98) | 0.36(0.29 to 0.43) |
| Andorra | 90(64 to 123) | 299(215 to 395) | 3077.29(2166.63 to 4177.56) | 3534.52(2542.14 to 4666.19) | 0.48(0.39 to 0.57) |
| Angola | 3169(2228 to 4325) | 9546(6650 to 12997) | 2401.49(1688.2 to 3277.7) | 2524.45(1758.59 to 3437.11) | 0.19(0.17 to 0.21) |
| Antigua and Barbuda | 33(23 to 45) | 58(40 to 78) | 912.5(641.72 to 1250.11) | 1094.9(760.2 to 1485.18) | 0.63(0.56 to 0.7) |
| Argentina | 69203(48945 to 94146) | 158184(113403 to 213165) | 3737.98(2643.75 to 5085.25) | 4625.17(3315.8 to 6232.76) | 0.75(0.69 to 0.81) |
| Armenia | 2014(1414 to 2745) | 4530(3200 to 6181) | 1750.59(1229.26 to 2386.14) | 2017.84(1425.55 to 2753.48) | 0.68(0.61 to 0.74) |
| Australia | 67003(48857 to 90380) | 235880(173928 to 314718) | 5537.73(4037.95 to 7469.81) | 8427.94(6214.42 to 11244.85) | 1.68(1.51 to 1.85) |
| Austria | 20413(14563 to 27716) | 40456(29384 to 53531) | 2672.67(1906.68 to 3628.79) | 3288.63(2388.59 to 4351.41) | 0.83(0.79 to 0.87) |
| Azerbaijan | 3820(2711 to 5155) | 6736(4642 to 9134) | 1724.83(1224.35 to 2328.03) | 2058.25(1418.49 to 2790.8) | 0.74(0.7 to 0.77) |
| Bahamas | 76(52 to 102) | 207(147 to 281) | 939.51(651.57 to 1273.53) | 1107.6(785.24 to 1501.7) | 0.59(0.56 to 0.63) |
| Bahrain | 148(103 to 202) | 652(453 to 885) | 2514.29(1756.93 to | 2790.53(1939.72 to | 0.2(0.15 to 0.25) |

| | | | | | |
|---|---|---|---|---|---|
| | | | 3442.51) | 3785.74) | |
| Bangladesh | 43628(31103 to 58947) | 140411(100115 to 190434) | 1958.77(1396.42 to 2646.53) | 2016.19(1437.57 to 2734.49) | 0.12(0.09 to 0.15) |
| Barbados | 200(138 to 275) | 342(241 to 458) | 934.82(646.11 to 1284.56) | 1123.96(793.11 to 1503.65) | 0.64(0.61 to 0.67) |
| Belarus | 12384(8737 to 16844) | 18073(12773 to 24486) | 1770.7(1249.2 to 2408.38) | 1939.45(1370.68 to 2627.66) | 0.43(0.37 to 0.48) |
| Belgium | 26819(19344 to 36184) | 50861(37319 to 67908) | 2788.02(2010.92 to 3761.64) | 3265.79(2396.28 to 4360.42) | 0.61(0.59 to 0.63) |
| Belize | 52(37 to 71) | 160(115 to 214) | 1001.03(706.88 to 1368.12) | 1243.08(891.28 to 1661.52) | 0.76(0.67 to 0.85) |
| Benin | 2398(1698 to 3244) | 5316(3683 to 7204) | 2328.94(1649.3 to 3150.6) | 2440.51(1690.94 to 3307.43) | 0.12(0.11 to 0.14) |
| Bermuda | 35(25 to 48) | 99(70 to 134) | 1014.84(716.43 to 1380.86) | 1166.2(830.84 to 1587.53) | 0.5(0.46 to 0.55) |
| Bhutan | 157(109 to 216) | 603(423 to 815) | 1714.94(1186.12 to 2350.8) | 2101.37(1473.82 to 2839.71) | 0.71(0.61 to 0.8) |
| Bolivia (Plurinational State of) | 1429(1005 to 1926) | 5420(3861 to 7410) | 946.17(665.05 to 1274.74) | 1192.62(849.54 to 1630.52) | 0.85(0.83 to 0.87) |
| Bosnia and Herzegovina | 2173(1524 to 2965) | 5260(3648 to 7218) | 1332.32(934.37 to 1817.97) | 1481.13(1027.25 to 2032.61) | 0.52(0.47 to 0.58) |
| Botswana | 622(434 to 854) | 1528(1064 to 2051) | 2507.94(1749.27 to 3444.02) | 2874.18(2000.75 to 3856.28) | 0.39(0.27 to 0.52) |
| Brazil | 40095(28020 to 54311) | 153876(110146 to 206353) | 947.85(662.39 to 1283.92) | 1175.66(841.55 to 1576.6) | 0.8(0.76 to 0.84) |
| Brunei Darussalam | 121(84 to 167) | 373(261 to 519) | 3268.48(2254.31 to 4505.58) | 3755.54(2632.9 to 5225.8) | 0.49(0.4 to 0.58) |
| Bulgaria | 9840(6712 to 13628) | 15808(11194 to 21678) | 1493.81(1019.01 to 2068.82) | 1535.69(1087.47 to 2105.89) | 0.13(0.12 to 0.15) |

| | | | | | |
|---|---|---|---|---|---|
| Burkina Faso | 4248(2987 to 5799) | 8930(6275 to 12108) | 2160.07(1518.76 to 2948.56) | 2283.6(1604.7 to 3096.35) | 0.17(0.12 to 0.21) |
| Burundi | 2749(1931 to 3757) | 4149(2921 to 5595) | 2395.68(1682.91 to 3273.25) | 2535.81(1785.43 to 3420.25) | 0.21(0.18 to 0.23) |
| Cabo Verde | 334(236 to 456) | 498(355 to 664) | 2200.09(1552.01 to 3001.14) | 2375.97(1694.99 to 3170.98) | 0.2(0.13 to 0.26) |
| Cambodia | 4347(3063 to 5850) | 13460(9342 to 18483) | 2394.13(1687.09 to 3221.79) | 2522.39(1750.67 to 3463.8) | 0.22(0.21 to 0.24) |
| Cameroon | 4576(3170 to 6227) | 12606(8740 to 17043) | 2481.03(1718.89 to 3376.28) | 2576.96(1786.7 to 3484.09) | 0.08(0.05 to 0.1) |
| Canada | 106353(76510 to 142993) | 319204(235929 to 426482) | 5287.95(3804.16 to 7109.73) | 7099.42(5247.29 to 9485.38) | 1.18(1.1 to 1.25) |
| Central African Republic | 895(625 to 1244) | 1515(1057 to 2047) | 2255.59(1574.4 to 3134.22) | 2249.6(1569.58 to 3039.55) | -0.04(-0.06 to -0.02) |
| Chad | 3307(2291 to 4498) | 5867(4129 to 7962) | 2192.86(1518.78 to 2982.06) | 2409.38(1695.76 to 3269.73) | 0.3(0.28 to 0.32) |
| Chile | 21036(15070 to 28572) | 68821(49002 to 92673) | 3942.43(2824.23 to 5354.61) | 4772.86(3398.39 to 6427) | 0.67(0.62 to 0.72) |
| China | 1014293(718148 to 1380843) | 4041719(2833617 to 5464267) | 2651.18(1877.11 to 3609.27) | 3743.55(2624.57 to 5061.15) | 1.66(1.42 to 1.91) |
| Colombia | 4488(3148 to 6170) | 20131(14475 to 27022) | 540.57(379.23 to 743.28) | 650.84(467.99 to 873.63) | 0.61(0.59 to 0.63) |
| Comoros | 288(201 to 387) | 647(460 to 867) | 2480.59(1732.15 to 3334.91) | 2549.61(1814.06 to 3419.44) | 0.14(0.12 to 0.16) |
| Congo | 1074(751 to 1459) | 2740(1918 to 3721) | 2429.01(1698.96 to 3299.49) | 2750.53(1924.78 to 3734.69) | 0.47(0.44 to 0.51) |
| Cook Islands | 23(16 to 31) | 61(43 to 84) | 3787.53(2651.21 to 5068.51) | 4418.38(3102.19 to 6011.14) | 0.39(0.29 to 0.49) |

| | | | | | |
|---|---|---|---|---|---|
| Costa Rica | 538(380 to 736) | 1935(1388 to 2625) | 566.06(399.97 to 774.47) | 672.09(482.04 to 912.09) | 0.65(0.6 to 0.71) |
| Côte d'Ivoire | 3244(2240 to 4456) | 9933(6969 to 13599) | 2428.29(1677 to 3335.24) | 2502.07(1755.58 to 3425.48) | 0.03(0.01 to 0.06) |
| Croatia | 4400(3080 to 6042) | 9091(6378 to 12264) | 1348.47(943.99 to 1851.59) | 1539.72(1080.22 to 2077.08) | 0.63(0.58 to 0.69) |
| Cuba | 5990(4187 to 8216) | 13402(9585 to 18051) | 950.05(664.01 to 1303.11) | 1106.01(791.05 to 1489.66) | 0.54(0.52 to 0.56) |
| Cyprus | 1461(1018 to 1992) | 4103(2936 to 5502) | 2817.61(1962.4 to 3841.28) | 3313.68(2371.67 to 4443.59) | 0.62(0.56 to 0.69) |
| Czechia | 10962(7661 to 14906) | 22875(16348 to 31319) | 1376.44(962 to 1871.67) | 1559.66(1114.67 to 2135.42) | 0.73(0.3 to 1.16) |
| Democratic People's Republic of Korea | 18911(13328 to 25588) | 58846(41408 to 78960) | 2974.29(2096.26 to 4024.55) | 3243.11(2282.1 to 4351.65) | 0.3(0.21 to 0.39) |
| Democratic Republic of the Congo | 14930(10353 to 20448) | 31485(21894 to 42745) | 2546.74(1766.08 to 3488.06) | 2342.92(1629.22 to 3180.85) | -0.34(-0.43 to -0.24) |
| Denmark | 15525(11096 to 20901) | 26643(19215 to 35498) | 2773.71(1982.46 to 3734.36) | 3246.53(2341.36 to 4325.53) | 0.59(0.56 to 0.63) |
| Djibouti | 102(71 to 141) | 603(418 to 829) | 2361.77(1646.15 to 3258.16) | 2885.58(2001.37 to 3966.76) | 0.72(0.68 to 0.75) |
| Dominica | 44(31 to 60) | 60(42 to 82) | 873.96(612.55 to 1194.82) | 1104.54(775.38 to 1516.78) | 0.82(0.76 to 0.88) |
| Dominican Republic | 1724(1203 to 2329) | 5719(4064 to 7714) | 927.17(646.63 to 1252.53) | 1132.87(805.08 to 1528.01) | 0.75(0.71 to 0.8) |
| Ecuador | 3105(2164 to 4211) | 12292(8968 to 16240) | 1141.73(795.45 to 1548.13) | 1460.92(1065.94 to 1930.18) | 0.89(0.85 to 0.94) |
| Egypt | 29598(20477 to 39852) | 73106(51396 to 99736) | 2329.52(1611.59 to 3136.52) | 2850.67(2004.11 to 3889.06) | 0.6(0.55 to 0.64) |

| | | | | | |
|---|---|---|---|---|---|
| El Salvador | 857(606 to 1163) | 2411(1721 to 3243) | 543.14(384.48 to 737.42) | 655.51(467.94 to 881.59) | 0.63(0.56 to 0.71) |
| Equatorial Guinea | 181(127 to 248) | 537(378 to 726) | 2276.33(1603.02 to 3117.39) | 2756.73(1941.3 to 3726.13) | 0.87(0.79 to 0.95) |
| Eritrea | 587(404 to 806) | 1934(1344 to 2624) | 2009.53(1382.31 to 2758.74) | 2200.85(1529.87 to 2985.8) | 0.31(0.28 to 0.33) |
| Estonia | 2132(1481 to 2892) | 3816(2720 to 5138) | 1796.82(1248.13 to 2436.92) | 2069.98(1475.32 to 2787.32) | 0.61(0.55 to 0.66) |
| Eswatini | 327(228 to 438) | 693(476 to 940) | 2613.54(1820.48 to 3498.88) | 2734(1876.97 to 3708.74) | 0.03(-0.11 to 0.16) |
| Ethiopia | 20186(13985 to 27610) | 54159(38223 to 72767) | 2625.05(1818.61 to 3590.51) | 2867.08(2023.45 to 3852.12) | 0.27(0.24 to 0.3) |
| Fiji | 476(331 to 646) | 1172(813 to 1583) | 3500.82(2437.81 to 4754.93) | 3842.04(2664.75 to 5188.43) | 0.27(0.24 to 0.3) |
| Finland | 11969(8508 to 15953) | 28852(20615 to 38590) | 2618.8(1861.54 to 3490.52) | 3265.78(2333.38 to 4367.93) | 0.84(0.81 to 0.88) |
| France | 144999(103274 to 194963) | 300374(218494 to 399207) | 2778.46(1978.92 to 3735.85) | 3181.74(2314.42 to 4228.65) | 0.57(0.54 to 0.61) |
| Gabon | 658(457 to 896) | 1210(841 to 1627) | 2460.61(1708.63 to 3348.74) | 2817.87(1957.34 to 3790) | 0.39(0.36 to 0.41) |
| Gambia | 352(248 to 484) | 1134(788 to 1528) | 2251.57(1585.24 to 3090.85) | 2434.37(1690.32 to 3279.06) | 0.19(0.16 to 0.22) |
| Georgia | 5704(3948 to 7738) | 7504(5319 to 9985) | 1792.12(1240.39 to 2431.27) | 2007.24(1422.77 to 2671.1) | 0.46(0.43 to 0.49) |
| Germany | 215404(155153 to 291558) | 446033(323269 to 590386) | 2666.12(1920.37 to 3608.7) | 3363.91(2438.04 to 4452.6) | 0.95(0.9 to 1) |
| Ghana | 5584(3925 to 7626) | 16067(11335 to 21740) | 2193.42(1541.83 to 2995.35) | 2356.9(1662.77 to 3188.97) | 0.21(0.17 to 0.24) |

| | | | | | |
|---|---|---|---|---|---|
| Greece | 29885(21299 to 40192) | 60896(44363 to 81444) | 3147.64(2243.37 to 4233.3) | 3546.46(2583.57 to 4743.09) | 1.48(0.62 to 2.35) |
| Greenland | 61(43 to 82) | 198(142 to 268) | 4717.61(3358.79 to 6385.41) | 6323.4(4541.02 to 8572.96) | 1.11(1.06 to 1.16) |
| Grenada | 45(32 to 63) | 59(41 to 81) | 893.77(634.2 to 1241.96) | 1005.07(697.26 to 1367.53) | 0.28(0.22 to 0.34) |
| Guam | 98(68 to 132) | 425(308 to 559) | 3519.47(2453.95 to 4744.59) | 4123.23(2983.53 to 5425.54) | 0.5(0.44 to 0.55) |
| Guatemala | 771(532 to 1062) | 3687(2624 to 5117) | 503.35(347.7 to 693.97) | 604.19(430.01 to 838.41) | 0.69(0.67 to 0.72) |
| Guinea | 3771(2636 to 5131) | 6312(4418 to 8591) | 2282.02(1595.49 to 3105.23) | 2429.06(1700.2 to 3305.94) | 0.23(0.18 to 0.28) |
| Guinea-Bissau | 393(270 to 541) | 613(427 to 826) | 2252.27(1551.07 to 3103.46) | 2191.2(1525.03 to 2951.93) | -0.18(-0.22 to -0.14) |
| Guyana | 158(111 to 215) | 284(198 to 385) | 902.35(632.78 to 1229.68) | 1042.23(726.03 to 1411.67) | 0.57(0.51 to 0.62) |
| Haiti | 1094(761 to 1515) | 2778(1968 to 3741) | 799.17(555.5 to 1105.89) | 950.72(673.5 to 1280.45) | 0.67(0.63 to 0.71) |
| Honduras | 516(362 to 703) | 1907(1353 to 2593) | 516.93(363.12 to 705.01) | 618.75(439.03 to 841.13) | 0.67(0.61 to 0.73) |
| Hungary | 12303(8541 to 16811) | 20033(14177 to 26963) | 1452.84(1008.51 to 1985.12) | 1535.15(1086.39 to 2066.16) | 0.24(0.21 to 0.26) |
| Iceland | 551(402 to 733) | 1249(908 to 1665) | 3010.12(2193.12 to 4000.77) | 3514.77(2554.99 to 4684.52) | 0.58(0.55 to 0.6) |
| India | 311960(219237 to 422909) | 1156386(818051 to 1553861) | 1908.01(1340.89 to 2586.59) | 2046.09(1447.44 to 2749.37) | 0.14(0.08 to 0.19) |
| Indonesia | 97916(69044 to 132193) | 271763(190766 to 364229) | 2568.71(1811.29 to 3467.94) | 3025.63(2123.87 to 4055.09) | 0.45(0.4 to 0.49) |

| | | | | | |
|---|---|---|---|---|---|
| Iran (Islamic Republic of) | 22070(15587 to 30330) | 90164(63047 to 122073) | 2297.12(1622.36 to 3156.81) | 2596.94(1815.91 to 3516) | 0.29(0.1 to 0.47) |
| Iraq | 9522(6679 to 12743) | 24393(17012 to 32639) | 2490.54(1746.92 to 3333.12) | 2511.9(1751.86 to 3361.05) | 0.02(0 to 0.03) |
| Ireland | 7781(5545 to 10436) | 16280(11927 to 21637) | 2896.69(2064.06 to 3884.87) | 3376.01(2473.24 to 4486.9) | 0.58(0.55 to 0.61) |
| Israel | 9073(6537 to 12345) | 24539(17978 to 32629) | 2978.64(2145.95 to 4052.6) | 3275.67(2399.92 to 4355.56) | 0.36(0.33 to 0.4) |
| Italy | 145079(102845 to 194532) | 301886(217609 to 401842) | 2619.23(1856.75 to 3512.04) | 2933.36(2114.46 to 3904.62) | 0.58(0.18 to 0.98) |
| Jamaica | 1078(762 to 1469) | 2021(1436 to 2733) | 926.36(655.16 to 1263.02) | 1144.42(813.02 to 1547.36) | 0.78(0.71 to 0.86) |
| Japan | 316753(220643 to 424649) | 1000088(721917 to 1328476) | 3204.28(2232.03 to 4295.75) | 3627.76(2618.71 to 4818.97) | 0.51(0.47 to 0.54) |
| Jordan | 1134(793 to 1533) | 7433(5237 to 10091) | 2320.11(1623.41 to 3136.13) | 2694.9(1898.59 to 3658.62) | 0.53(0.46 to 0.6) |
| Kazakhstan | 10689(7491 to 14477) | 15337(10736 to 20847) | 1783.09(1249.6 to 2415.07) | 1996.63(1397.64 to 2713.89) | 0.44(0.42 to 0.47) |
| Kenya | 10042(7078 to 13580) | 24318(17258 to 32517) | 2715.26(1913.78 to 3671.81) | 2747.53(1949.85 to 3673.88) | 0(-0.1 to 0.11) |
| Kiribati | 41(29 to 57) | 74(52 to 101) | 3002.4(2099.86 to 4123.19) | 3315.79(2318 to 4503.59) | 0.3(0.22 to 0.38) |
| Kuwait | 545(388 to 731) | 3044(2178 to 4086) | 2700.74(1921.98 to 3622.04) | 3252.85(2327.47 to 4365.9) | 1.22(1.02 to 1.43) |
| Kyrgyzstan | 2603(1831 to 3569) | 3475(2429 to 4687) | 1692.73(1190.76 to 2320.91) | 1913.09(1337.22 to 2580.17) | 0.47(0.45 to 0.49) |
| Lao People's Democratic Republic | 2258(1590 to 3078) | 5211(3623 to 7075) | 2519.11(1774.08 to 3434.61) | 2893.69(2011.9 to 3928.95) | 0.49(0.48 to 0.51) |

| | | | | | |
|---|---|---|---|---|---|
| Latvia | 3814(2635 to 5251) | 5672(4016 to 7704) | 1834.12(1266.9 to 2524.77) | 2016.24(1427.44 to 2738.36) | 0.45(0.37 to 0.52) |
| Lebanon | 2363(1650 to 3175) | 8388(5980 to 11274) | 2332.69(1628.95 to 3134.51) | 2617.09(1865.84 to 3517.76) | 0.38(0.31 to 0.44) |
| Lesotho | 1071(742 to 1439) | 1361(939 to 1843) | 2293.75(1588.04 to 3081.52) | 2499.11(1723.19 to 3383.41) | 0.23(0.2 to 0.27) |
| Liberia | 1421(998 to 1929) | 2196(1540 to 2967) | 2433.68(1708.03 to 3303.4) | 2604.72(1825.93 to 3519.02) | 0.29(0.24 to 0.34) |
| Libya | 2160(1525 to 2918) | 6345(4502 to 8607) | 2522.49(1780.91 to 3407.58) | 2728.97(1936.12 to 3701.8) | 0.28(0.25 to 0.31) |
| Lithuania | 4847(3363 to 6616) | 7944(5682 to 10825) | 1901.62(1319.34 to 2595.74) | 1985.46(1420.19 to 2705.53) | 0.26(0.2 to 0.31) |
| Luxembourg | 961(681 to 1307) | 2164(1566 to 2887) | 2807.39(1990.47 to 3820.71) | 3437.55(2488.37 to 4587.02) | 0.78(0.75 to 0.81) |
| Madagascar | 5785(4038 to 7805) | 9268(6449 to 12544) | 2585.33(1804.91 to 3488.13) | 2499.5(1739.27 to 3382.98) | -0.12(-0.16 to -0.08) |
| Malawi | 3775(2637 to 5127) | 7764(5373 to 10488) | 2371.71(1657.18 to 3221.65) | 2419.46(1674.26 to 3268.43) | 0.13(0.1 to 0.17) |
| Malaysia | 11815(8329 to 15937) | 43329(30358 to 58547) | 2810.61(1981.2 to 3791.12) | 3381.14(2368.94 to 4568.6) | 0.73(0.67 to 0.79) |
| Maldives | 90(64 to 123) | 422(298 to 577) | 3011.9(2130.89 to 4115.75) | 3265.39(2307.02 to 4470.59) | 0.37(0.3 to 0.43) |
| Mali | 3842(2643 to 5181) | 9009(6359 to 11986) | 2174.91(1496.41 to 2933.37) | 2400.1(1694.2 to 3193.27) | 0.36(0.33 to 0.39) |
| Malta | 723(520 to 985) | 2216(1610 to 2966) | 2890.26(2080.62 to 3940.63) | 3362.41(2443.39 to 4501.86) | 0.54(0.5 to 0.57) |
| Marshall Islands | 23(16 to 32) | 41(29 to 56) | 3081.64(2152.57 to 4238.98) | 3739.93(2621.54 to 5090.57) | 0.68(0.6 to 0.76) |

| | | | | | |
|---|---|---|---|---|---|
| Mauritania | 1224(852 to 1669) | 2767(1914 to 3713) | 2333.61(1624.97 to 3181.92) | 2748.51(1900.71 to 3687.66) | 0.52(0.46 to 0.58) |
| Mauritius | 896(636 to 1202) | 2792(1979 to 3749) | 2642.76(1875.84 to 3546.02) | 3084.56(2185.78 to 4141.74) | 0.56(0.54 to 0.58) |
| Mexico | 14908(10419 to 20458) | 50022(35222 to 67793) | 710.22(496.36 to 974.58) | 785.36(552.99 to 1064.37) | 0.79(0.52 to 1.07) |
| Micronesia (Federated States of) | 68(48 to 93) | 81(56 to 110) | 3326.62(2336.83 to 4544.52) | 3576.84(2451.22 to 4839.45) | 0.1(0.02 to 0.19) |
| Monaco | 148(106 to 198) | 239(174 to 319) | 2966.75(2137.68 to 3980.94) | 3427.02(2496.04 to 4569.39) | 0.54(0.48 to 0.61) |
| Mongolia | 943(649 to 1285) | 1607(1124 to 2209) | 1828.7(1257.29 to 2491.15) | 1921.7(1343.78 to 2640.96) | 0.2(0.15 to 0.26) |
| Montenegro | 472(335 to 641) | 894(623 to 1227) | 1478.03(1050.15 to 2007.46) | 1542.35(1074.18 to 2116.91) | 0.25(0.21 to 0.29) |
| Morocco | 13602(9443 to 18396) | 33843(23520 to 45678) | 2183.78(1516.08 to 2953.33) | 2444.79(1699.06 to 3299.73) | 0.37(0.35 to 0.39) |
| Mozambique | 5720(4005 to 7846) | 10645(7468 to 14250) | 2333.77(1633.77 to 3201.09) | 2373.87(1665.44 to 3177.81) | 0.02(0 to 0.05) |
| Myanmar | 24488(17281 to 33692) | 56480(39665 to 76309) | 2469.32(1742.62 to 3397.49) | 2682.84(1884.09 to 3624.71) | 0.36(0.33 to 0.39) |
| Namibia | 974(678 to 1330) | 1872(1326 to 2556) | 2590.12(1802.24 to 3537.18) | 2719.1(1925 to 3710.97) | 0.15(0.12 to 0.19) |
| Nauru | 4(3 to 5) | 3(2 to 4) | 3609.11(2530.87 to 4922.85) | 3693.69(2567.65 to 5096.67) | 0.45(0.22 to 0.68) |
| Nepal | 6716(4643 to 9136) | 21495(14910 to 29400) | 1846.63(1276.56 to 2512.14) | 1964.23(1362.53 to 2686.67) | 0.3(0.26 to 0.34) |
| Netherlands | 37620(27306 to 50578) | 80648(59570 to 108232) | 2927.54(2124.92 to 3935.93) | 3462.26(2557.35 to 4646.42) | 0.19(0.03 to 0.35) |

| | | | | | |
|---|---|---|---|---|---|
| New Zealand | 18056(12821 to 24301) | 46106(35928 to 57896) | 7297.87(5181.84 to 9822.11) | 8893.74(6930.33 to 11168.03) | 0.49(0.38 to 0.61) |
| Nicaragua | 379(265 to 516) | 1332(945 to 1816) | 517.9(361.47 to 705.14) | 618.93(439.11 to 843.73) | 0.59(0.49 to 0.68) |
| Niger | 2310(1605 to 3147) | 7029(4866 to 9472) | 2232.96(1551.63 to 3042.62) | 2265.32(1568.24 to 3052.57) | -0.02(-0.08 to 0.03) |
| Nigeria | 47676(33479 to 63844) | 82334(58209 to 110807) | 2329.51(1635.83 to 3119.48) | 2331.73(1648.5 to 3138.1) | -0.13(-0.4 to 0.13) |
| Niue | 5(3 to 6) | 5(3 to 7) | 3280.47(2336.64 to 4436.19) | 3913.23(2737.71 to 5262.37) | 0.58(0.45 to 0.71) |
| North Macedonia | 1319(928 to 1825) | 2754(1927 to 3760) | 1508.93(1061.62 to 2087.73) | 1540.25(1077.99 to 2103.44) | 0.14(0.09 to 0.2) |
| Northern Mariana Islands | 19(13 to 26) | 64(45 to 86) | 3824.83(2646.93 to 5242.01) | 4055.13(2854.3 to 5442.8) | 0.11(0.01 to 0.2) |
| Norway | 12445(8865 to 16763) | 19507(14107 to 26145) | 2547.19(1814.48 to 3430.91) | 2991.75(2163.57 to 4009.83) | 0.06(-0.16 to 0.27) |
| Oman | 472(329 to 648) | 1456(1017 to 1980) | 2053.12(1431.5 to 2815.86) | 2702.5(1887.24 to 3673.84) | 1.01(0.9 to 1.12) |
| Pakistan | 51266(35868 to 70251) | 95349(66738 to 130486) | 1738.83(1216.56 to 2382.76) | 2109.46(1476.49 to 2886.83) | 0.92(0.75 to 1.09) |
| Palau | 17(12 to 24) | 33(23 to 45) | 3655.35(2527.96 to 4955.17) | 4083.72(2865.16 to 5529.9) | 0.26(0.14 to 0.38) |
| Palestine | 956(676 to 1299) | 2220(1551 to 3016) | 2273.38(1606.88 to 3087.71) | 2311.96(1615.62 to 3140.97) | -0.05(-0.1 to 0) |
| Panama | 424(303 to 577) | 1620(1142 to 2199) | 518.83(371.43 to 706.43) | 661.75(466.59 to 898.22) | 0.77(0.74 to 0.8) |
| Papua New Guinea | 2023(1409 to 2773) | 5560(3909 to 7635) | 3098.43(2158.03 to 4247.08) | 3386.76(2381.09 to 4650.67) | 0.32(0.3 to 0.34) |

| | | | | | |
|---|---|---|---|---|---|
| Paraguay | 1055(752 to 1458) | 3126(2244 to 4181) | 907.95(647.14 to 1254.76) | 1077.69(773.71 to 1441.48) | 0.63(0.58 to 0.67) |
| Peru | 6247(4457 to 8273) | 23868(16949 to 32111) | 1045.06(745.62 to 1384.01) | 1292.58(917.91 to 1738.98) | 0.81(0.78 to 0.84) |
| Philippines | 30204(21279 to 40878) | 95819(67308 to 128979) | 2313.55(1629.95 to 3131.16) | 2846.1(1999.24 to 3831.06) | 0.77(0.62 to 0.93) |
| Poland | 35104(24799 to 47882) | 72504(51567 to 96814) | 1456.8(1029.16 to 1987.07) | 1647.59(1171.8 to 2200) | 0.51(0.48 to 0.54) |
| Portugal | 24421(17455 to 33051) | 56482(40970 to 75534) | 2830.29(2022.97 to 3830.47) | 3312.97(2403.14 to 4430.48) | 0.66(0.6 to 0.72) |
| Puerto Rico | 2432(1716 to 3277) | 6520(4745 to 8777) | 1081.12(762.84 to 1456.99) | 1285.38(935.4 to 1730.25) | 0.69(0.64 to 0.75) |
| Qatar | 65(45 to 89) | 524(359 to 731) | 2711.34(1889.01 to 3720.04) | 3444.82(2356.4 to 4805.71) | 0.7(0.58 to 0.81) |
| Republic of Korea | 37847(26499 to 51696) | 182529(129178 to 249263) | 2977.88(2085 to 4067.49) | 3474.92(2459.24 to 4745.37) | 0.55(0.53 to 0.58) |
| Republic of Moldova | 3774(2648 to 5070) | 6616(4634 to 8941) | 1789.85(1255.87 to 2404.18) | 2056.13(1440.29 to 2778.74) | 0.62(0.53 to 0.71) |
| Romania | 20345(14033 to 27933) | 38456(27437 to 52424) | 1420.16(979.6 to 1949.91) | 1568.43(1119.02 to 2138.12) | 0.48(0.43 to 0.52) |
| Russian Federation | 168729(118159 to 228594) | 288863(203898 to 387575) | 1749.2(1224.94 to 2369.81) | 2119.97(1496.41 to 2844.41) | 0.79(0.75 to 0.83) |
| Rwanda | 2886(2004 to 3984) | 5467(3844 to 7391) | 2353.44(1634.2 to 3249) | 2338.39(1644.3 to 3161.24) | -0.03(-0.09 to 0.03) |
| Saint Kitts and Nevis | 24(17 to 33) | 30(21 to 41) | 911.77(630.13 to 1242.33) | 1126.09(799.66 to 1529.16) | 0.78(0.7 to 0.86) |
| Saint Lucia | 46(32 to 64) | 135(95 to 185) | 880.14(604.72 to 1211.58) | 1098.6(768.36 to 1503.7) | 0.82(0.76 to 0.87) |

| | | | | | |
|---|---|---|---|---|---|
| Saint Vincent and the Grenadines | 38(26 to 52) | 84(60 to 115) | 860.95(598.31 to 1181.67) | 1121.13(795.03 to 1531.91) | 1(0.97 to 1.04) |
| Samoa | 147(103 to 197) | 268(189 to 365) | 3565.83(2496.01 to 4788.04) | 3947.76(2793.89 to 5388.4) | 0.28(0.24 to 0.32) |
| San Marino | 64(46 to 88) | 153(112 to 203) | 2979.26(2114.5 to 4083.08) | 3435.59(2519.53 to 4580.16) | 0.5(0.47 to 0.52) |
| Sao Tome and Principe | 75(52 to 102) | 113(79 to 154) | 2261.4(1577.25 to 3067.21) | 2539.64(1771.33 to 3452) | 0.37(0.32 to 0.42) |
| Saudi Arabia | 6155(4298 to 8246) | 15106(10575 to 20439) | 2504.43(1748.73 to 3355.3) | 3105.16(2173.65 to 4201.42) | 0.72(0.65 to 0.78) |
| Senegal | 3647(2567 to 4989) | 8437(5895 to 11361) | 2411.15(1697.12 to 3298.55) | 2437.6(1703.22 to 3282.42) | -0.04(-0.07 to -0.01) |
| Serbia | 7735(5506 to 10298) | 16422(11561 to 22596) | 1468.06(1044.95 to 1954.58) | 1596.77(1124.08 to 2197.11) | 0.32(0.29 to 0.35) |
| Seychelles | 92(66 to 124) | 163(115 to 219) | 2691.56(1915.06 to 3599.81) | 3098.93(2193.22 to 4178.13) | 0.45(0.4 to 0.5) |
| Sierra Leone | 2379(1665 to 3272) | 3930(2716 to 5327) | 2327.18(1628.81 to 3201.41) | 2377.69(1643.31 to 3223.2) | 0.01(-0.02 to 0.05) |
| Singapore | 3396(2378 to 4642) | 15976(11222 to 21552) | 3244.65(2271.46 to 4434.71) | 3822.64(2685.13 to 5156.9) | 0.54(0.51 to 0.57) |
| Slovakia | 4819(3365 to 6546) | 8579(6034 to 11605) | 1458.12(1018.11 to 1980.4) | 1525.35(1072.93 to 2063.49) | 0.17(0.14 to 0.2) |
| Slovenia | 1865(1283 to 2561) | 4589(3276 to 6177) | 1373.79(945.05 to 1886.51) | 1597.88(1140.48 to 2150.78) | 0.65(0.58 to 0.71) |
| Solomon Islands | 148(105 to 204) | 367(258 to 501) | 3489.83(2461.53 to 4792.62) | 3569.68(2512.75 to 4865.79) | 0.06(-0.01 to 0.12) |
| Somalia | 1942(1353 to 2624) | 5051(3525 to 6916) | 2389.69(1664.81 to 3229.26) | 2220.19(1549.54 to 3039.88) | -0.21(-0.27 to -0.14) |

| | | | | | |
|---|---|---|---|---|---|
| South Africa | 30488(21655 to 40632) | 66901(47146 to 89618) | 2961.29(2103.3 to 3946.6) | 3097.24(2182.68 to 4148.94) | 0.15(0.08 to 0.21) |
| South Sudan | 2822(1967 to 3830) | 4172(2929 to 5610) | 2509.05(1748.82 to 3406.07) | 2712.44(1904.24 to 3647.39) | 0.31(0.24 to 0.37) |
| Spain | 99507(71250 to 133648) | 220186(159994 to 291671) | 2887.54(2067.58 to 3878.27) | 3322.73(2414.39 to 4401.47) | 0.46(0.41 to 0.5) |
| Sri Lanka | 13608(9432 to 18466) | 38320(26846 to 51714) | 2739.89(1899.04 to 3717.99) | 2811.94(1969.99 to 3794.84) | 0.13(0.1 to 0.15) |
| Sudan | 9851(6887 to 13616) | 21700(15123 to 29328) | 2093.11(1463.34 to 2893.11) | 2588.03(1803.64 to 3497.77) | 0.7(0.68 to 0.71) |
| Suriname | 124(87 to 168) | 351(249 to 474) | 959.8(677.16 to 1302.11) | 1113.68(791.13 to 1504.34) | 0.57(0.54 to 0.59) |
| Sweden | 33589(23741 to 45337) | 55885(39858 to 74375) | 3074.26(2172.87 to 4149.5) | 3633.95(2591.78 to 4836.29) | 0.68(0.59 to 0.77) |
| Switzerland | 19407(13981 to 25995) | 38145(27919 to 50689) | 2779.95(2002.81 to 3723.68) | 3190.08(2334.85 to 4239.15) | 0.51(0.48 to 0.53) |
| Syrian Arab Republic | 5455(3854 to 7297) | 13683(9615 to 18608) | 2434.58(1720.26 to 3256.83) | 2560.34(1799.17 to 3481.86) | 0.07(0.01 to 0.14) |
| Taiwan (Province of China) | 27820(20381 to 38124) | 105072(78522 to 136362) | 3810.03(2791.21 to 5221.2) | 4690.93(3505.58 to 6087.84) | 0.63(-0.3 to 1.57) |
| Tajikistan | 2494(1784 to 3372) | 2766(1932 to 3760) | 1784.98(1276.86 to 2413.62) | 1926.8(1345.51 to 2619.19) | 0.4(0.36 to 0.44) |
| Thailand | 40208(29718 to 53802) | 181851(129259 to 244427) | 2629.73(1943.64 to 3518.77) | 3214.76(2285.04 to 4320.99) | 1.18(1 to 1.35) |
| Timor-Leste | 225(158 to 305) | 1087(756 to 1463) | 2606.67(1831.37 to 3523.01) | 2749.6(1913.06 to 3702.4) | 0.15(0.13 to 0.17) |
| Togo | 1145(811 to 1552) | 2920(2027 to 3989) | 2224.07(1576.39 to 3014.5) | 2077.58(1442.06 to 2837.54) | -0.34(-0.38 to -0.3) |

| | | | | | |
|---|---|---|---|---|---|
| Tokelau | 3(2 to 4) | 3(2 to 4) | 3443.8(2421.51 to 4645.59) | 4146.85(2920.56 to 5554.73) | 0.64(0.6 to 0.69) |
| Tonga | 93(66 to 127) | 170(120 to 228) | 3560.99(2513.34 to 4826.07) | 3854.04(2724.83 to 5164.2) | 0.16(0.06 to 0.25) |
| Trinidad and Tobago | 446(310 to 609) | 1179(830 to 1609) | 944.41(657.07 to 1291.04) | 1129.51(795.52 to 1541.57) | 0.68(0.6 to 0.76) |
| Tunisia | 5600(3909 to 7684) | 16057(11407 to 21532) | 2301.66(1606.62 to 3158.11) | 2448.03(1739.12 to 3282.78) | 0.18(0.17 to 0.19) |
| Türkiye | 36881(26130 to 49913) | 121764(86454 to 163876) | 2322.24(1645.3 to 3142.8) | 2547.03(1808.43 to 3427.92) | 0.24(0.16 to 0.32) |
| Turkmenistan | 1487(1060 to 2009) | 3117(2228 to 4175) | 1753.99(1250.25 to 2369.55) | 2063.06(1474.88 to 2763.65) | 0.65(0.62 to 0.69) |
| Tuvalu | 9(6 to 12) | 18(13 to 25) | 2995.41(2112.4 to 4018.24) | 3674.46(2536.94 to 4953.89) | 0.61(0.49 to 0.74) |
| Uganda | 7095(4920 to 9774) | 14245(10071 to 19159) | 2398.46(1663.23 to 3304.18) | 2389.38(1689.29 to 3213.47) | 0.03(-0.01 to 0.08) |
| Ukraine | 73218(51169 to 99257) | 96200(67742 to 130687) | 1826.93(1276.76 to 2476.63) | 2039.86(1436.43 to 2771.14) | 0.48(0.44 to 0.52) |
| United Arab Emirates | 236(164 to 325) | 1235(844 to 1707) | 2500.97(1733.1 to 3444.3) | 3062.42(2092.15 to 4233.3) | 0.9(0.81 to 0.99) |
| United Kingdom | 177455(126970 to 238241) | 308661(224683 to 412565) | 2855.78(2043.32 to 3834) | 3516(2559.4 to 4699.58) | 1.57(1.33 to 1.81) |
| United Republic of Tanzania | 12233(8558 to 16421) | 29666(21050 to 40133) | 2486(1739.25 to 3337.21) | 2708.35(1921.73 to 3663.95) | 0.27(0.23 to 0.31) |
| United States of America | 966343(699336 to 1286862) | 3059118(2470181 to 3703064) | 4528.07(3276.93 to 6029.95) | 8508.06(6870.1 to 10299.01) | 3.55(2.93 to 4.18) |
| United States Virgin Islands | 42(29 to 57) | 143(100 to 191) | 1022.97(712.12 to 1391.41) | 1155.16(808.58 to 1548.22) | 0.42(0.34 to 0.49) |

| | | | | | |
|---|---|---|---|---|---|
| Uruguay | 9280(6568 to 12509) | 17120(12352 to 22896) | 3778.74(2674.38 to 5093.41) | 4639.23(3347.1 to 6204.37) | 0.71(0.66 to 0.77) |
| Uzbekistan | 9437(6756 to 12665) | 10659(7426 to 14650) | 1744.6(1248.92 to 2341.35) | 1947.33(1356.56 to 2676.34) | 0.48(0.44 to 0.52) |
| Vanuatu | 99(69 to 134) | 298(210 to 409) | 3562.28(2459.45 to 4827.68) | 3751.25(2637.69 to 5138.64) | 0.15(0.14 to 0.16) |
| Venezuela (Bolivarian Republic of) | 2615(1847 to 3589) | 10110(7281 to 13665) | 550.12(388.65 to 755.09) | 661.13(476.09 to 893.58) | 0.63(0.61 to 0.64) |
| Viet Nam | 46153(32710 to 62269) | 102717(73068 to 139598) | 2265.09(1605.34 to 3056.06) | 2491.42(1772.29 to 3385.97) | 0.57(0.47 to 0.66) |
| Yemen | 3449(2434 to 4691) | 12478(8800 to 17022) | 1774.57(1251.98 to 2413.17) | 2207.01(1556.41 to 3010.6) | 0.82(0.75 to 0.88) |
| Zambia | 3153(2169 to 4303) | 7187(5032 to 9755) | 2712.82(1866.23 to 3702.75) | 2613.12(1829.54 to 3546.94) | -0.23(-0.27 to -0.19) |
| Zimbabwe | 4677(3196 to 6331) | 7397(5163 to 10068) | 2606.38(1780.71 to 3528.17) | 2555.63(1783.91 to 3478.34) | -0.13(-0.17 to -0.1) |
